# Expert Views on Therapeutic Climbing—A Multi-Perspective, Qualitative Study

**DOI:** 10.3390/ijerph18073535

**Published:** 2021-03-29

**Authors:** Anika Frühauf, Julia Heußner, Martin Niedermeier, Martin Kopp

**Affiliations:** Department of Sport Science, University of Innsbruck, 6020 Innsbruck, Austria; julia.heussner@hotmail.com (J.H.); martin.niedermeier@uibk.ac.at (M.N.); Martin.Kopp@uibk.ac.at (M.K.)

**Keywords:** exercise therapy, attitudes, expert views, mental health

## Abstract

Therapeutic climbing (TC) is regularly used as an add-on treatment option for a variety of disorders. However, evidence on the assessment of professionals deciding on the treatment options or assessing the appropriateness of treatment options is lacking. Therefore, the aim of the present study was to assess the potential of TC as an exercise intervention from different expert perspectives. The study was designed as a qualitative, problem-centered interview experiment to assess the perspectives of pedagogues, physicians, and psychologists on exercise therapy in general and the potential of TC. The sample consisted of 30 experts (10 pedagogues, 10 physicians, and 10 psychologists) with different levels of experience in TC (ᴓage: 41.7 years, ♀ = 43%). Overall, the potential of TC as an add-on treatment option for various disorders was rated by the respondents as positive and useful. The interviewed experts believed that TC can have a decisive effect on the social, psychological, and physiological domain as a sensibly used add-on therapy. However, considering the reported potential adverse effects and the costs connected with TC, it should not be considered as a panacea. Although research in this area is still much in its infancy, the positive perspective of the professional representatives surveyed could facilitate access to TC for patients and may foster more research in this field.

## 1. Introduction

Exercise is associated with reduced mortality risk and has the potential to influence mental health positively [1]. Alongside its preventive effect as well as its intervention effect for many somatic disorders, exercise can also be used as a low-threshold mental health intervention strategy that is highly efficient, cost effective, and with few side effects [2]. Although exercise has been shown to be effective in the treatment of mental health disorders [3,4,5], there is not enough information about potential differing effects between different modalities of exercise interventions and different doses of specific activities [5]. Although exercise interventions have been predominantly conducted using aerobic exercise as an intervention method, it seems that resistance and mixed training might even show larger effects on mood than aerobic training alone [3,5].

One of these exercise modalities used in the treatment of depression and various disorders is therapeutic climbing (TC). Consisting of elements of resistance and whole-body strength endurance training [6,7], TC also contains unique socio-psychological characteristics [8]. TC can be organized in different forms, i.e., bouldering horizontally without a rope up to a maximal height possible to jump on a mat [9] or climbing using a rope and a harness belayed by a climbing partner [8]. Using TC as a treatment for mental health disorders, psychotherapy was reported to be integrated in climbing sessions [9,10]. This included among others, mindfulness exercise at the beginning and end of each session, as well as reflecting on climbing experiences and the integration of these measures in everyday life [10]. TC is increasingly finding its way into clinical treatment; however, empirical evidence is not yet clear [6]. From a physiological point of view, preliminary studies assessing the physiological effects of TC groups showed increased muscle strength, balance, and mobility [7]. Climbing has potential positive effects on coordination, strength, and core tension [11] and is concomitantly associated with a low injury risk (0.02 injuries per 1000 h in indoor climbing) [12]. Aside from the physiological benefits, TC provides potential desirable psychological effects. Perhaps as a consequence of an increasing popularity of climbing, TC has been used for health-enhancing exercise interventions and has recently been evaluated as successful in reducing depressive symptoms and improving psychological well-being in clinical populations [9,10]. In a recent study, our group investigated different physical activities in children and adolescents during in-patient treatment for mental health disorders; TC as well as swimming were effective in positively changing affective responses. In addition, TC might have a beneficial impact on affective valence, especially in the first half of the climbing session [13]. Furthermore, through its unique demands, involving unusual heights as well as responsibility and trust in the belaying partner, climbing might enhance concentration, focus, and psychosocial aspects such as cooperation and respect, and trigger emotions, such as fear, joy, or pride [11]. Achievements can be reached by adapting height or difficulty individually, and improvements are easily visible for patients (i.e., by reaching the top of a route or getting the next hold) [11,14]. Further, competitiveness between participants is believed to be less important compared to team ball sports [14]. All these aspects may address autonomy, competence, and relatedness, which might, based on self-determination theory in combination with activation and valence, enhance intrinsic motivation [15]. Despite all these potential positive aspects, more data from randomized controlled trials are needed to investigate whether or not TC might be a modality of exercise intervention that improves the named physiological and psychological aspects and shows higher or different health-enhancing effects than other exercise interventions [6].

Nevertheless, climbing is increasingly established in therapeutic settings in order to positively affect physiological and especially psychosocial health domains [6,16]. In the phase of increased use as a health intervention, an essential aspect is to record the attitude and outcome expectancies toward this intervention of professionally involved groups. The previous literature has described that physicians′ attitudes and expectations of outcomes regarding exercise have a significant impact on prescribing behavior [17,18]. The expectations of healthcare providers even play an important role in the treatment outcome [19]. To verify the conditions for the systematic integration of a therapeutic intervention into the healthcare system, it seems important to know more about attitudes and expectations regarding TC and regarding exercise in general, which might influence the attitude toward TC of the professionals who are working in healthcare or education.

To the best of our knowledge, there are no reports on the attitudes and expectations of different professional groups toward climbing as a form of therapy. For this reason, this paper aims to shed light on the following questions: (1) What are the attitudes of health experts from different fields toward the use of exercise in general and specifically therapeutic climbing as an add-on treatment option? (2) What are the domains where therapeutic climbing is expected to result in improved health-enhancing outcomes? (3) What should be considered when establishing TC as a treatment option?

## 2. Materials and Methods

### 2.1. Design and Procedure

Since the aim of the study was to explore rather than to validate pre-existing hypotheses, a qualitative approach was selected. A semi-structured interview was carried out with each participant. An interview guide was used to ensure that each participant was asked the same questions but at the same time was allowed to talk freely about their experiences. The interview guide was developed and agreed upon by the research team following the guidelines of Weischer [20]. As an introductory question, experts were asked about their theoretical and practical knowledge of TC. If participants did not report any knowledge of TC, the following information was given to them: “A prerequisite for therapy is the recognition of a disease from where a form of therapy is derived. Therapeutic climbing is usually an adjunctive therapy and is already used in the field of physiotherapy, occupational therapy, and psychotherapy. In occupational therapy and psychotherapy, the patient communicates with the therapist in a secure setting with the help of a third medium, the climbing wall. This is done in a combination of experiencing climbing and speaking about the experiences, with the goal of individually treating the patient.”

Next, six open questions were asked about the attitude toward exercise therapy in general (What is your opinion on exercise therapy?) and further about the attitude and effectiveness of therapeutic climbing (e.g., How do you estimate the effectiveness of TC? In which areas would you expect TC to be effective? Do you think TC could be associated with negative effects? In which areas would you consider TC to be a useful therapy method?). If answers were short, interviewees were asked to further elaborate on the topic or give examples. The last question related to factors associated with establishing TC as a treatment option. The average interview duration was between 30 and 40 min. All interviews were conducted and analyzed in German and were carried out one-to-one.

The interview questions were structured into attitudes toward the effectiveness of exercise therapies, theoretical and practical knowledge in the field of TC, attitudes toward the effectiveness of therapeutic climbing, assumptions of potential effects of TC, and considerations when establishing TC as a treatment option.

### 2.2. Participants

Since data saturation is expected after 20–30 interviews [21], 30 experts from three different professional groups (medicine, education, psychology) were interviewed. The selection of these professional groups was based on the rationale that the approaches of exercise therapy are anchored in both medicine and psychology. The third group of pedagogues was selected to assess the socio-psychological potential of TC and appropriateness of exercise/TC from an educational perspective.

When selecting the interview partners, attention was paid to ensuring that the average age and gender distribution were similar between the groups. Ten persons from each professional group were interviewed. The study period covered approximately two months, and the selection of the interview partners was done by a snowball system starting with two known specialists in each group [22].

The group of physicians comprised general practitioners, orthopedists, neurologists, and psychiatrists, and the pedagogues came from the fields of social pedagogy, curative and special education, and teaching. The psychologists worked in the field of occupational and organizational psychology, and behavioral and clinical psychology, and five were trained as psychotherapists. Further information about demographics and experience with TC can be seen in Table 1. Participants were informed about the study procedure when contacting them prior to the interviews verbally. Informed consent was signed by each participant at the interview. The study was in line with the guidelines of conducting surveys approved by the Board for Ethical Questions in Science of the University of Innsbruck in accordance with the Declaration of Helsinki.

### 2.3. Analyses

Before analyzing the data, all interviews were transcribed verbatim; transcription was carried out immediately after each interview. Phrases connected to the research questions were highlighted, and any non-verbal communications were noted. Data were then analyzed using thematic content analysis [23] in several distinct stages. First, transcripts were read several times to get immersed in the data. Second, a thematic content analysis was carried out where raw data were given codes (e.g., concentration). This procedure was repeated for all 30 interviews. In the next step of the analysis, all interviews were cross-checked, ensuring that coding was consistent and accurately represented the data. Following this, similar codes were grouped into themes and, if necessary, for a better understanding into subthemes (see Table 1). The final step was to confirm the codes and main categories with the research team. In terms of disagreement or uncertainty by the primary analyst, codes and main themes were discussed until full agreement was reached. Interview excerpts, which serve as supporting documents, are each marked Med.1 to Med.10 for medical physicians, Psy.1 to Psy.10 for psychologists, and Ped.1 to Ped.10 for pedagogues.

## 3. Results

Three themes resulted from the analysis: attitudes toward exercise therapy in general, expected effects of therapeutic climbing, and aspects related to the establishment of therapeutic climbing (Table 2). Overall, the majority in all three expert groups had a positive attitude toward exercise and climbing therapy. Although approximately only half of the experts had practical experience with TC, they expected TC as being effective mostly in the psychological area but also in social and physiological areas. They were further worried about potential adverse effects of TC on patients. Each group saw the highest applicability of TC in their specific occupational field. All groups reported the need for a multi-professional team in rehabilitation and the need for a specialist to prescribe TC. Since only marginal differences between expert groups occurred, differences between groups were only mentioned if applicable.

### 3.1. Attitude toward Exercise Therapy in General

Overall, 28 of the 30 respondents showed similar positive attitudes toward exercise therapies (not limited to TC) in the form of an accompanying therapy with conventional medical (e.g., medication, surgery) or psychological therapies. All but two respondents (both physicians) considered exercise as additional therapy as useful and helpful. Some further described exercise as an alternative to medication and mentioned the importance of individual treatment options for patients, as explained by one pedagogue:

“Therapy must be individually adapted to the patient. Every patient has a different clinical picture, and different individual demands of therapy, and from the therapist′s point of view, these should be met. What is enriching for one patient is a waste of time for another.”

(Ped.9)

The medical experts explained the usefulness of exercise therapy as following: “Exercise therapy alone can sometimes be sufficient, for example, for regeneration after surgery.”

(Med.7)

“(...) this [exercise] activates the body′s hormonal system, and the self-healing powers can be improved.”

(Med.10)

Although Med.7 stated the effectiveness of exercise as a stand-alone therapy, most of the respondents saw exercise as an accompanying therapy in an overall treatment concept. It was further mentioned that exercise therapy can promote motor skills such as balance, endurance, and strength.

Those physicians who were skeptical about the effectiveness of exercise therapy mentioned financial aspects and the importance of the primary healing method (e.g., surgery), as explained below:

“(...) The question is who pays for something like that? Surgery without additional therapy would not be possible, but first and foremost, the surgery is the important step. […] The effectiveness of surgery is only supported by additional therapies. However, all these [exercise] therapies have an advantage compared to pharmaceuticals—they do not harm!”

(Med.1)

The effectiveness of exercise therapy in the form of an accompanying therapy was rated as effective by all 10 psychologists, especially in the treatment of behavioral disorders and attention deficit disorders.

The 10 interviewed pedagogues also considered additional therapy in the form of exercise as effective and health promoting and had a positive attitude. Both psychological and pedagogical experts mentioned the importance of a multi-professional team that decided together on the best therapy options that should complement each other.

Aspects of alternative versus pharmaceutical interventions were frequently mentioned. Seven of the interviewees made a comparison between exercise therapy and medication. Three physicians stated that for some diseases, exercise therapy alone may also be sufficient. As examples, orthopedic injuries or physiological rehabilitation were named.

“I consider it more useful to do exercise therapy than medication. Sometimes medication can′t help, but exercise can.”.

(Med.7)

The majority of the interviewed pedagogues and psychologists, but not the physicians, had the opinion that medication was prescribed too often in the first place.

### 3.2. Expected Effects of Therapeutic Climbing

The effects of therapeutic climbing were categorized into four subthemes: psychological effects (including cognitive effects), physiological effects, social effects, and adverse effects. The interviewed experts mostly had a positive attitude toward therapeutic climbing, regardless of whether they already had had experience with TC. They expected health-enhancing effects related to psychological, social, and physiological changes. Possible adverse effects were also mentioned, however, less frequently. Health-enhancing effects were expected in all three domains. The only notable difference between groups appeared in the applicability of TC (Table 3). Psychologists and physicians saw high applicability in the psychological domain, whereas pedagogues saw the highest applicability of TC in the educational domain. Using TC in the educational domain was only mentioned by less than half of physicians and psychologists. Using TC in physiotherapy settings was suggested by 90% of the physicians but only by four psychologists and three pedagogues.

Overall, in none of the analyzed categories, a difference was detected in the statements between those respondents who practiced therapeutic climbing compared to those who did not actively use it.

In the following section, the individual subthemes are explained in more detail and are underlined by quotes from the different occupational groups.

#### 3.2.1. Psychological Effects

Psychological effects through TC were expected by the majority of experts, as this psychologist explains: “There is a fundamental change in mental state initiated by climbing.” (Psy.4).

Within the psychological effects, the following individual codes were found: self-esteem, fears, and limits, as well as focus and motivation. Interviewed experts mentioned that they expected changes in self-esteem through climbing that could be possibly transferred to everyday life, like one psychologist and one pedagogue explained: “Climbing therapy is a good tool in my experience; it can help to increase self-worth and possibly increase the experience with self-efficacy.” (Psy.4) “Perhaps the self-awareness can be transformed into the private or school domain.” (Ped.6) Fears and limits were seen as an opportunity to confront the patient’s fears and to overcome them in a safe and guided environment, like a pedagogue and psychologist explained: “The patients learn to overcome their fear, to let go and let themselves go.” (Ped.1) “Fears can be confronted in a conscious and controlled way and with guidance improvements are possible.” (Psy.8) They further thought that through the specific psychological and physiological demands in climbing, patients had to focus on the specific task and thus attention and concentration might be enhanced. This could be especially useful for children with attention deficit disorders.

“When climbing, you have high [physiological and psychological] demands that you have to concentrate on. You get direct feedback when you are not concentrated.”

(Ped.7)

“You consciously think about (...) can I reach there now or rather somewhere else, (...) what do I do next? I think that in climbing, you take steps consciously.”

(Ped.2)

“When I think of children with attention deficit disorder, I can imagine climbing therapy being helpful. Here they have to show full concentration and attention.”

(Ped. 9)

Especially physicians talked about a higher therapy adherence in TC because it might enhance participants’ motivation and increase well-being. “Some clients in my climbing therapy show so much joy that they also go climbing in their free time. My experience shows that climbing has a domino effect.” (Med.8)

“Climbing has a high emotional and motivational character.”

(Med.8)

#### 3.2.2. Social Effects

Social effects were mainly expected in the form of enhancing trust and communication. The element of trust is mentioned as a basic factor of TC. Both practicing and non-practicing climbing therapists addressed this aspect. In total, 25 of the interviewees commented on this topic. They explained how the belaying part of the climbing activity is a fundamental part of building trust and confidence. “You can build on this basis of confidence and continue to work.” (Psy.7) However, it was mentioned that the belayer should be careful not to abuse the trust of the climber.

“Patients think rationally and weigh up what they can and cannot trust themselves to do. If they have a certain amount of confidence in themselves, they come out of therapy stronger.”

(Ped.3)

Climbing was further seen as a valuable tool for shaping relationships. By experiencing and speaking with each other, climbing therapy pursued a holistic approach. The relaxed atmosphere offered the possibility of a relaxed communication, like this pedagogue explained: “I imagine that the patient does not have to talk as much as in a conventional session. Through the experience, communication runs all by itself.” (Ped.7)

#### 3.2.3. Physiological Effects

Experts, especially physicians, spoke of expected physiological effects in terms of enhanced coordination, muscle growth, and the interplay between tension and relaxation.

Enhanced coordination was explained by a physician as follows: “Climbing is a holistic movement with an enormous driving function. There is an improvement in the coordinative and conditional abilities through the climbing itself. Most patients are also motivated by climbing to do sports in their free time.” (Med.9) Another physician expected muscle growth in the upper extremities. “Especially in the area of the upper extremities, muscle growth can be expected.” (Med.10) “Since the patient is usually subject to other forms of therapy, muscle growth cannot be attributed to climbing therapy alone, but maybe due to other therapies. Such as targeted strength and rehabilitation training after a surgery.” (Med.7) A psychophysiological effect of the interplay between tension while climbing and having overcome the task and experiencing relaxation was seen as beneficial, as explained here: “The phases of tension and relaxation alternate. Before and during the climb, you can often see the tension on the patient′s face. Signs of this, according to some therapists, are tense facial features and a stiff posture. After the patients have reached their goal and are back on the ground, signs of relaxation become apparent. The closer they get to the ground and thus into their ‘comfort zone,’ the more relaxed they appear.” (Psy.7)

#### 3.2.4. Adverse Effects

Possible adverse effects were described by experts from all groups. Specifically, experts were worried about overtaxing patients and inexperienced therapists and to a lesser extent about pain and injuries through TC. Only two psychologists using TC as a therapy method mentioned no adverse effects of climbing therapy. Overtaxing was described through excessive fear and ambitious patients, as follows: “There are performance-oriented, ambitious patients who show sensation-seeking behavior. They want great effects and are then quickly disappointed [if they do not reach their expectations]. These patients have to be hold back.” (Psy.6) “A bad experience at an altitude may result in fear and a discontinuation of climbing therapy.” (Psy.10) Physicians were worried about pain and injuries that might even be manifested in the patient. “Pain in the hands and feet can also result from shoes that are too tight, which affects the patient′s motivation. In addition, overexerting, both on a psychological and on a somatic level, is a possible negative consequence, which can manifest itself in a form of anxiety.” (Med.5) To avoid negative consequences like overtaxing, pain, and injuries, the importance of a trained therapist in TC was frequently mentioned.

“The therapist must be well trained so that no negative effects can occur. Good and individual guidance is important. Furthermore, the therapist has the task of creating a sense of achievement so that climbing therapy does not bring any negative consequences.” (Psy.4) “It must be well guided therapeutically, under supervision. Otherwise, it is dangerous. It is a fine line, but this is the art of the therapist to use his knowledge and skills wisely.”

(Med.4)

### 3.3. Aspects Related to the Establishment of Therapeutic Climbing

Prescribing TC was mostly named in connection with a specialist and a multi-professional team. However, there was high uncertainty concerning prescription possibilities, and the financial aspect was mentioned frequently.

When talking about prescription, many respondents from the groups of psychologists and pedagogues hesitated with their answers, indicating a great deal of uncertainty, but also verbally expressed it. Five of the interviewed psychologists and pedagogues indicated too little knowledge about prescribing climbing therapy. No uncertainty arose among the interviewed medical professionals. They all mentioned that the prescription should either come from a physician (provided a diagnosis has been made) or a specialist. As specialists, doctors, psychotherapists, and physiotherapists were named throughout the expert groups. All expert groups stated that a possible prescription for climbing therapy should come from a specialist. However, most of the interviewees through all groups considered consultation with a multi-professional team to be important.

“It would be easiest if doctors, as well as psychotherapists and occupational therapists, had the possibility to prescribe within their multi-professional team.”

(Ped.5)

Financial aspects were named as a problem in TC. Psychologists and pedagogues were all in favor of reimbursement of TC through the national health insurance system. Two doctors were more critical:

“In our system, prescriptions are the responsibility of doctors as soon as they cost money. Physiotherapists can see an indication for a special therapy and pass this on.”

(Med.1)

“Nowadays, people like to have recreational activities such as wellness or massages prescribed, even though they don’t need them.”

(Med.10)

The most important question would be a normal billing within the framework of a generally prescribed exercise therapy.

Pedagogues also emphasized that the respective specialist should have the authority to prescribe a specific form of therapy.

“I am a therapist and have been doing climbing with mentally ill people since 2011. For a year now, there has been occupational therapy climbing on prescription. The practice is billed to the health insurance fund.”

(Psy.4)

As a future prognosis of TC, the majority of experts stated that they considered the potential of climbing therapy to be high. In addition, 24 of the 30 respondents thought that climbing therapy would continue to be popular. The importance of further research in this area was reinforced by the majority of interviewed experts (80%).

Medical professionals and psychologists saw potential in TC and believed that it will continue to become popular (“There I am not worried that it will establish itself permanently as a form of therapy.” (Psy.2)) but also talked about the necessity of fostering research in this area, as this physician explained:

“I believe that very specific forms of therapy have a future. It [therapy] is still becoming more specific (...). Maybe it is not a widely applicable form of therapy, but in special cases, it might become popular. If there are more studies on it, I see an upward trend in this field.”

(Med.8)

Among the interviewed pedagogues, six were in favor of climbing therapy in the future, but think that it should be used in combination with other therapies, as this pedagogue reported: “It is conceivable that in combination with physiotherapeutic or psychotherapeutic measures, there is a prospect for climbing therapy.” (Ped.8) The other respondents indicated too little knowledge in this area and did not give a clear prognosis.

## 4. Discussion

In the present study, attitudes and expectations toward therapeutic climbing from the perspective of three professional groups were investigated by semi-structured interviews. The majority of the interviewed experts considered exercise therapies in general and TC in particular as an enrichment for the healthcare system. Experts expected health-enhancing effects through TC in psychological, physiological, and social domains. Possible adverse effects, especially when TC is not conducted by a specialist, were mentioned. The necessity of a multi-professional team was frequently addressed as well as the financial aspects when prescribing TC.

### 4.1. Exercise as an Add-On Treatment Option

The vast majority of the sample had a positive attitude toward exercise in the treatment of various disorders. The effectiveness of exercise therapy was already evaluated in a number of different chronic diseases [3,5]. Many of the interviewees had their own experience in the field of exercise therapies and were interested in the topic. Given the importance of the professional groups interviewed in the healthcare system (i.e., for prescribing therapy and for conducting therapy as well as for educational purposes), it is desirable that a consciousness for the importance of exercise is present [18]. Based on the aspects mentioned, the following aspects seem to be important for the usage of exercise in general as an add-on treatment option. Exercise preferences, intensity, and durations vary between people, and this should be considered besides the modality of exercise [24]. The majority considered exercise as an add-on therapy to be useful. However, some experts stated that medication was administered too often. There is some evidence in people with mild–moderate depressive disorder that exercise is similarly effective compared to medical treatment [3,4]. Exercise could be used as a low-threshold intervention, especially in patients with mental health disorders [2]. However, it has to be mentioned critically that despite a large amount of evidence of the beneficial effects of exercise in various disorders, it is relatively rarely prescribed, with only a third of the people consulting a physician reported to have received exercise counseling in the U.S. [25]. Although exercise habits are an important predictor in exercise counseling and the current study revealed a positive attitude of experts toward exercise therapy in general and TC in particular, this does not seem to be the decisive reason for prescribing exercise. Among others, a lack of time and a lack of reimbursement were stated as barriers among doctors [25]. Whereas most studies investigating exercise counseling were conducted in the U.S. and other developed countries, the financial aspect was named frequently in the current study in both the context of prescription and as an adverse effect of TC.

### 4.2. The Potential of Therapeutic Climbing in the Treatment of Various Disorders

According to Ajzen [26], an attitude that is based on real behavioral experience is more accessible and consistent. Although only half of the participants had knowledge about TC, it was evaluated by all expert groups to be effective and health promoting, with a large range of possible application fields in psychotherapy, physiotherapy, and occupational therapy. Individually adapted to the needs of the participants, great effectiveness was expected. Overall, the experts of all three groups saw great potential in TC to achieve therapeutic success in a variety of domains.

In the psychological domain, expected benefits were related to self-esteem, the overcoming of fear, increased focus, and motivation. These aspects are important in many disorders but are seen as crucial in people with mental health disorders. Consequently, quantitative research on the evaluation of TC as an intervention method for people with mental health disorders has increased in the past 10 years [6]. A recent study showed that a 10-week TC program was superior in improving self-esteem scores and decreasing symptom severity compared to both waitlist control and home-based supervised exercise in patients with depressive disorder [9]. Climbing has a highly challenging character [11], which might stimulate arousal and, combined with positive reinforcement by success, pleasure [27]. Increase in the positive affect was shown in previous studies evaluating the acute effects of TC [8,13]. These acute effects are assumed as a potential mechanism for beneficial changes compared to other exercise modalities. In addition to expected benefits of self-esteem and motivation, pedagogues expected increased attention through TC and saw potential health-enhancing effects, especially for children with attention deficit hyperactivity disorder (ADHD). These expectations are supported by a preliminary finding in the literature. A case report by Lee and Song [28] supports this by reporting improved regulation of arousal and increased attention in children diagnosed with ADHD through therapeutic climbing, but the authors stated that TC cannot be applied to all children with ADHD.

Although listed in the physiological domain, the interplay between tension and relaxation falls between the physiological and the psychological domain. Although tension and relaxation are related to a physical sensation, both states are closely related to the psychological terms of activation and deactivation [29]. Connected with the dimension of pleasure, the expected effect is important in the acute emotion regulation of people with mental disorders [13]. Additional expected physiological improvements included coordinative aspects and muscle growth in the upper extremity, which is partially confirmed by the literature [7]. Not mentioned by the present sample but reported in the existing literature are muscular improvements in the backs in people with spine disorders [7,30]. However, the evidence level has to be considered as low, given the paucity of research in this field [7].

In the social domain, some experts expected an increase in social competence and social integration when providing TC in small groups. Most studies on therapeutic climbing have indicated group sessions of about 10 people [8,9]. Although to the best of our knowledge, studies on the potential effects of TC on social competences do not exist, the context of belaying another person during TC may provide a promising trigger for changes in the social domain. In the future, it might be interesting to investigate whether climbing therapy in an individual or group setting promotes different social aspects.

Possible adverse effects of TC were mentioned by 28 of the respondents, i.e., falling, injury to hands and feet, as well as a possible overtaxing of patients. However, indoor climbing is associated with a low injury risk [12]. More skepticism and uncertainty among experts were aroused about the financial aspects. Two medical professionals considered surgery and drug treatment to be the top priority in a healthcare system. The economic costs of TC (e.g., equipment, facility) seem to be higher than in standard exercise therapy [6]. A recognition of TC as a therapy form and paid prescription for TC are unlikely without comprehensive study results and thus are not expected in the near future. As many reports in the existing literature on therapeutic climbing do not reflect potential negative effects, the critical aspects, which are named as financial aspects, possible overtaxing of patients, the deterrent effect of pain and injuries, and the factor of an untrained therapist, should be considered and further evaluated. Especially, before being introduced as a funded health intervention, further evidence of positive and potential adverse effects seems crucial. This is also partly reflected by the responses of the present sample on the aspects related to the establishment of therapeutic climbing. Although most of the interviewees showed an affinity toward TC and saw a potential for the future, there seems to be a great need to clarify prescription possibilities and responsibilities. Especially pedagogical and psychological experts reported too little background knowledge in this area and were unsure about the responsibility of a prescription. However, prescribing exercise should be the role of the physician, who should have the necessary background and training to prescribe and promote exercise [31], although exercise prescription and promotion seem to be related to personal habits and preferences [25]. Analogous with the current state of research [6], the majority of interviewed experts stated that the research evidence in the field of therapeutic climbing is too small to prescribe TC yet. The affinity for TC was present regardless of whether they had already practiced this therapy form or whether this intervention was new to them. In none of the analyzed categories was a difference detected between the respondents with or without experience in the field of TC.

In summary, some of the expected health-enhancing effects of TC are supported by few earlier studies, especially psychological effects on people with mental disorders [6,8,9,10,13]. In other domains (e.g., physiological or social domain), empirical evidence is lacking. The data at this stage of research are not yet comprehensive enough to consequently promote TC as superior to other therapeutic exercise interventions [6]. It is recommended that future studies in the field consider all aspects of TC, including adverse (e.g., financial aspects, overtaxing, pain) and potential benefits (e.g., in the psychological, social, and physiological domains).

### 4.3. Strengths and Limitations

The most important strengths of this study are that it (1) investigated different professional groups with different experiences in the use of TC and (2) had a relatively large sample size. A qualitative approach was used for this study since qualitative research is often used to examine issues in great detail and depth [32]. However, readers should avoid generalizing the results of this study, because the selection of participants could be subject to a selection bias due to the chosen procedure. In addition, cultural and geographical factors may play a major role in the results achieved, as the popularity of climbing as a sport is probably above average in alpine regions. Most studies investigating the effects of TC were conducted in German-speaking regions [8,9,10,13] but are not limited to those. Scattered studies have been conducted in Denmark [14], Korea [28], and the U.S. [33].

## 5. Conclusions

This study aimed to investigate the attitudes and expectations regarding TC from the perspective of three professional groups working in the health sector. Overall, the potential of TC as an additional therapy was generally assessed as positive and useful by the experts. It is assumed that climbing therapy can have decisive effects on the social, psychological, and physiological areas as a sensibly used additional therapy, but this form of therapy should not be considered as a panacea for a wide range of health issues, and future study is warranted.

## Figures and Tables

**Table 1:** Participant description.

	Mean Age	Sex		Experience with Therapeutic Climbing	
	(Years)	Female	Male	Theory	Practice
Physicians	43.3	50%	50%	20%	20%
Psychologists	40.4	50%	50%	30%	30%
Pedagogues	41.5	30%	70%	90%	70%
Total	41.7	43%	57%	47%	40%

**Table 2:** Themes, subthemes, and codes.

Themes	Subthemes	Codes
Attitude toward exercise therapy in general		Effectiveness
		Supplement
		Alternative to medication
		Individuality
Expected effects of therapeutic climbing	Psychological	Self-esteem
		Fears and limits
		Focus
		Motivation
	Social	Trust
		Social component
		Competences
		Communication
	Physiological	Coordinative effects
		Muscle growth
		Interplay between tension and relaxation
	Adverse effects	Overtaxing
		Pain/Injuries
		Risks connected to unexperienced therapist
		Costs
Aspects related to the establishment of therapeutic climbing	Prescription	Uncertainty
		Specialist
		Financial aspect
		Multi-professional team
		State of research

**Table 3:** Estimated applicability of therapeutic climbing by expert groups.

	Psychological Domain	Physiological Domain	Social Domain
Physicians	90%	90%	40%
Psychologists	100%	40%	40%
Pedagogues	60%	30%	80%

## Data Availability

The data are not publicly available due to ethical considerations on preserving the anonymity of study participants.
